# Pigmented villonodular synovitis in pediatric population: review of literature and a case report

**DOI:** 10.1186/s12969-018-0222-4

**Published:** 2018-01-17

**Authors:** Mohsen Karami, Mehryar Soleimani, Reza Shiari

**Affiliations:** Mofid Children Hospital, Shahid Beheshti University of Medical Sciences, Shariati Ave., Tehran, 15514-15468 Iran

**Keywords:** Juvenile idiopathic arthritis, Patellar dislocation, Pigmented villonodular synovitis

## Abstract

**Background:**

Pigmented villonodular synovitis (PVNS) is a rare proliferative process in children that mostly affects the knee joint.

**Case Presentation:**

The study follows the case of a 3-year-old boy presenting recurrent patellar dislocation and PVNS. Due to symptoms such as chronic arthritis, he had been taking prednisolone and methotrexate for 6 months before receiving a definitive diagnosis. After a period of showing no improvements from his treatment, he was referred to our center and was diagnosed with local PVNS using magnetic resonance imaging (MRI). The patient was treated for his patellar dislocation by way of open synovectomy, lateral retinacular release, and a proximal realignment procedure, with no recurrence after a 24-month follow-up.

**Conclusion:**

PVNS may appear with symptoms resembling juvenile idiopathic arthritis, thus the disease should be considered in differential diagnosis of any inflammatory arthritis in children. PVNS may also cause mechanical symptoms such as patellar dislocation. In addition to synovectomy, a realignment procedure can be a useful method of treatment.

## Background

Pigmented villonodular synovitis (PVNS) is a rare proliferative process that affects the synovial joint, tendon sheaths, and bursa membranes [1]. The estimated incidence of PVNS is around 1.8 cases per million people in a population [1]. PVNS is usually found in adults aged 20–50 years, but it may also be found in children [2–4].

The youngest patient reported with PVNS was 12 months old [5]. Knee involvement is common in pediatric PVNS, although other affected areas have been reported, such as the foot and ankle, elbow, hip, sacroiliac joint, and multiple joint involvements [3, 5–32]. Some authors have determined that there is no sex predilection in PVNS, while other reports have shown a male or female predominance [1, 2, 33].

The etiology of PVNS is unknown, but chronic inflammation, a tumor-like disorder with chromosomal aberrations that cause hemorrhagic tendencies, as well as genetic factors, have been proposed as potential causes [2, 3]. Trauma and rheumatoid arthritis association have also been considered [14, 15, 33].

The following study reports on a 3-year-old boy with a 4-month-long history of pain and swelling in the right knee. The patient was initially treated for juvenile idiopathic arthritis (JIA) and growth retardation.

## Case Presentation

This study follows the case of a 3-year-old boy with a 4-month-long history of pain and swelling in the right knee and no history of trauma. The patient had been taking prednisolone and methotrexate for a period of 6 months in response to signs and symptoms very similar to juvenile idiopathic arthritis (JIA). Previously, he had also received a yearlong growth hormone treatment, for which the medical reason is unknown. He had no familial history of rheumatological conditions.

**Fig. 1:**
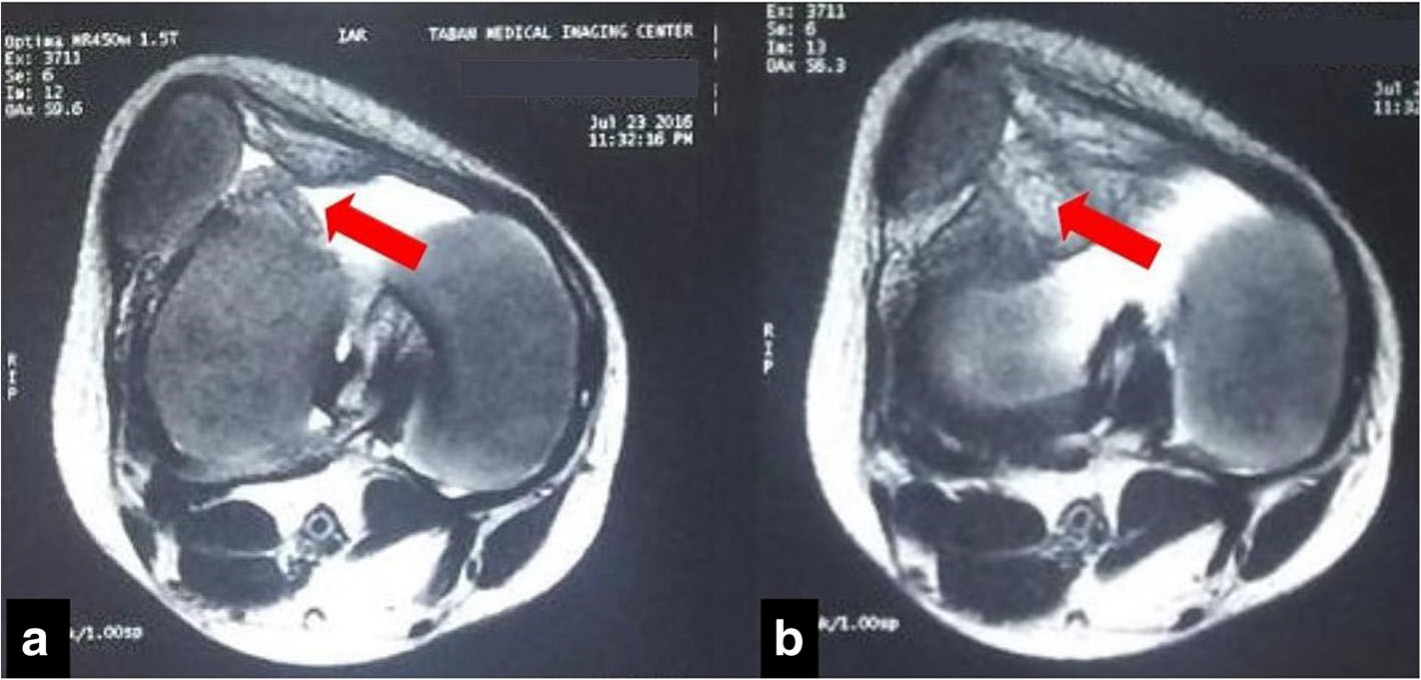
MR image. **a** Arrow showes synovial proliferation on T2-Weighted image. Note patellar dislocation and joint effusion. **b** Arrow indicates scattered low-signal intensity areas in the synovial membrane, representing hemosiderin deposits on T2-weighted images

A physical examination of the patient revealed swelling and a limited range of motion in the knee. It was clinically evident that the patella had been dislocated. The results of laboratory tests did not suggest that the patient suffered from JIA. Radiography images depicted soft tissue swelling around the knee joint. An MRI showed a large signal mass of synovium posterior to the lateral patellar facet, which caused lateral dislocation of the patella (Fig. 1).

**Fig. 2:**
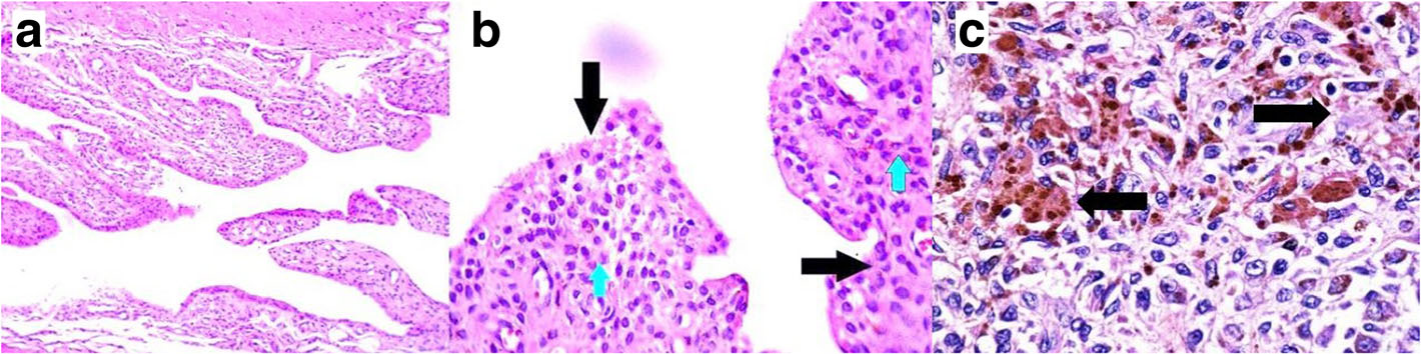
Microscopic section of the synovial biopsy, Pigmented Villonodular Synovitis, **a.** Villous and nodular configuration. Note the hypertrophied synovium. (×4 and ×10, Hematoxylin-Eosin), **b**. Hypertrophied synovium (black arrows) and histiocytes, Hemosiderin-laden macrophages and osteoclast-like giant cells (blue arrowheads) are visible. (×40, Hematoxylin-Eosin), **c**. This section shows admixture of hemosiderin-laden and lipid-laden macrophages

The chosen method of treatment for the patient was surgery, consisting of an open excisional biopsy and a proximal realignment procedure (vastus medialis obliquus advancement) through medial parapatellar incision. A histologic examination of the tissue exposed hyperplasia of the synovium, foamy macrophage, spindle cells, multinucleated giant cells and a considerable amount of hemosiderin deposits, all of which supported the diagnosis of PVNS (Fig. 2).

After the open surgical synovectomy and 3 months of physiotherapy, the swelling of the knee lessened and the range of motion in the knee returned to normal. The patient was followed for 24 months without any signs of recurrence.

## Discussion

This study reviews and summarizes 29 articles, published between 1975 and 2016, in which 42 cases of pediatric PVNS were reported [3, 5–32] (Table 1). Because of the impossibility to retrieve pediatric data, the articles representing mixed adult and pediatric cases were excluded from the review [34]. The mean age of the reported cases was 8.9 years. Sixty-two percent (*n* = 26) was female and 38% (*n* = 16) was male. PVNS affected only one joint in all but 6 cases, and the main localization was the knee joint (*n* = 23, 64%).

**Table 1:** Clinical presentations reported in literatures

Clinical presentation	Number of patients	Age (years)	Sex	Treatment (OS^a^/AS^b^)	Follow up	Recurrence	Authors
Knee pain and swelling	21	1–15 (mean 9.1)	10 M^c^, 11 F^d^	12 OS, 7 AS, 1 OS + AS, 1 injection	5 month-2 years	None	Baroni [3], Neubauer [12], Bruns [11], Jawadi [5], Deepak [14], Hong [6], Saulsburg [7], Jha [9], Maric [15], Nizam [13]
Painless swelling of knee	1	5	1 M	AS	2 years	None	Van Emelen [10]
Politeal cyst	1	12	1 M	OS + AS	–	–	Meehan [8]
Foot and ankle pain and swelling	4	3–14 (mean 10.3)	3 M 1 F	OS	6 month-2 years	None	Neubauer [12], Aghasi [24], Freedman [23], Kaneko [26]
Painless mass of foot and ankle	3	11–12 (mean 11.7)	1 M 2 F	OS	1–4 years	None	Zambakides [25], Sofier [21], Duncan [22]
Elbow	1	8	1 F	AS	–	–	Hsiu Su [19]
Non tender mass or swelling of elbow	2	6 (mean 6)	2 F	OS	2 years	None	Sekiya [18], Aydingoz [17]
Multifocal involvement	6	4.5–7 (mean 5.8)	1 M 5 F	OS	6 month-25 years	3 cases	Mukhopadhyay [28], Tavangar [27], Vedantam [29], Zhao [30], Walls [32], Leszczynski [31]
Hip pain	1	7	1 F	AS + OS	–	Not reported	Higuchi [20]
Sacral joint involvement	1	12	1 F	OS	–	–	Kang [20]
Hand swelling	1	7	1 F	OS	7 year	1 case	Neubauer [12]
Average	3.8	8.9	F/M: 1.6/1	OS 69%, AS 21.5%, OS + AS 7%, Injection 2.5	4.9 years	9.5%	

^a^Open synovectomy

^b^Arthroscopic synovectomy

^c^Male

^d^Female

Approximately 81% (*n* = 34) of patients presented joint pain and swelling. This entity may have been misdiagnosed with other causes of arthritis such as rheumatoid arthritis, hemophilic arthropathy, tuberculosis, and neoplastic processes [3, 15].

A localized intra-articular form of PVNS that causes mechanical symptoms, such as anterior knee pain, meniscal injury, and patellar dislocation has been reported in adult PVNS [35–39]. Mechanical symptoms are extremely rare in pediatric cases of PVNS [3, 5–32]. Recurrent patellar dislocation is a rare presentation of PVNS that was previously only reported in adult cases [38]. This study presents a 3-year-old boy with patellar dislocation caused by PVNS, an unusual demonstration of PVNS at an unusual age. To the best of our knowledge, this is the first report of patellar dislocation in pediatric PVNS.

Joint aspiration fluid in PVNS is usually serosanguinous and, without a history of trauma, can be nearly diagnostic [2]. Initial stages of radiography may show an increasing density or radiolucent defect with a thin sclerotic rim [2, 3]. Only 33% of reported cases of pediatric PVNS produced positive findings on the initial X-ray [3]. In the present case, radiography images showed nothing more than swelling of the soft tissue. CT-scans are useful in determining the severity of bone loss in large joints such as the hip and knee. Medium-contrast enhanced CT-scans may also be worthwhile in detecting recurrent lesions [2]. The most effective screening method for diagnosis of PVNS is the MRI. MRIs can reveal scattered low-signal intensity areas in the synovial membrane, representing hemosiderin deposits on T2-weighted images, and dotted areas of low-signal intensity, representing fibrous components of the lesion on T1-weighted images [1–3] (Fig. 1).

There is not a standard method of treatment for PVNS, especially in pediatric patients [1–3]. The aim of treatment for PVNS is to remove all abnormal tissue in order to relieve pain, lower the risk of joint destruction, and avoid local recurrence. The localized form of PVNS tends to be episodic and result in a good prognosis, while the diffuse form tends to progress gradually and recur more frequently [1, 4, 33]. Though there are several therapeutic solutions, the most common treatment is surgery [3]. Open or arthroscopic subtotal synovectomy is the most widely accepted surgery [1–3]. Some authors prefer arthroscopy to treat the localized form because this method results in less morbidity and a similar outcome as open surgery [1]. Some others prefer open surgery to arthroscopy because of the latter’s disadvantages, such as the risk of recurrence and the possibility of portal contamination [3].

The literature reported 3 recurrences in cases in which the large joints were affected in multifocal form, and one recurrence in a case affecting the small hand joints (Table 1). The present case was treated with open synovectomy, lateral release, and a proximal realignment procedure and did not show any signs of recurrence during the 24 months following the treatment.

Radiotherapy and isotopic synoviorthesis have been used in adults for relapse cases or as adjuvant modality, but the use of radiotherapy in children is controversial, due to the possibility of post-irradiation sarcoma and damage to the physis [1–4]. Because PVNS, or tenosynovial giant cell tumor (TGCT), has characteristic cytogenetic abnormalities resulting in the overexpression of colony stimulating factor 1 (CSF1), systemic medication targeting the CSF1/CSF1R axis (imatinib, nilotinib, emactuzumab, and PLX3397) has been proposed in patients with diffuse, relapsed, or multifocal PVNS/TGCT [40]. The results from related studies will need to be confirmed in larger, ideally randomized clinical trials [41].

## Conclusion

Although PVNS is a rare condition in pediatric patients, it can occur with symptoms resembling juvenile idiopathic arthritis, therefore it should be considered in differential diagnosis of any arthritis in children. PVNS in children may cause mechanical symptoms such as patellar dislocation. In these cases, lateral release and a proximal realignment procedure, in addition to synovectomy, can be useful methods of treatment.

## Abbreviations

JIA: Juvenile idiopathic arthritis
MRI: Magnetic resonance imaging
PVNS: Pigmented villonodular synovitis
VMO: Vastus Medialis Obliquus
